# Complex Susceptibilities and Chiroptical Effects of Collagen Measured with Polarimetric Second-Harmonic Generation Microscopy

**DOI:** 10.1038/s41598-019-48636-w

**Published:** 2019-08-28

**Authors:** Ahmad Golaraei, Lukas Kontenis, Kamdin Mirsanaye, Serguei Krouglov, Margarete K. Akens, Brian C. Wilson, Virginijus Barzda

**Affiliations:** 1 Princess Margaret Cancer Centre, University Health Network, Toronto, M5G 1L7 Canada; 2 University of Toronto, Department of Physics, Toronto, M5S 1A7 Canada; 3 University of Toronto Mississauga, Department of Chemical and Physical Sciences, Mississauga, L5L 1C6 Canada; 4 Light Conversion Ltd., LT-10223 Vilnius, Lithuania; 5 Vilnius University, Laser Research Centre, Faculty of Physics, Vilnius, 10223 Lithuania; 6 Techna Institute, University Health Network, Toronto, M5G 1L5 Canada; 7 University of Toronto, Department of Surgery, Toronto, M5S 1A1 Canada; 8 University of Toronto, Department of Medical Biophysics, Toronto, M5G 1L7 Canada

**Keywords:** Biophysics, Biological physics, Nonlinear optics

## Abstract

Nonlinear optical properties of collagen type-I are investigated in thin tissue sections of pig tendon as a research model using a complete polarimetric second-harmonic generation (P-SHG) microscopy technique called double Stokes-Mueller polarimetry (DSMP). Three complex-valued molecular susceptibility tensor component ratios are extracted. A significant retardance is observed between the chiral susceptibility component and the achiral components, while the achiral components appear to be in phase with each other. The DSMP formalism and microscopy measurements are further used to explain and experimentally validate the conditions required for SHG circular dichroism (SHG-CD) of collagen to occur. The SHG-CD can be observed with the microscope when: (i) the chiral second-order susceptibility tensor component has a non-zero value, (ii) a phase retardance is present between the chiral and achiral components of the second-order susceptibility tensor and (iii) the collagen fibres are tilted out of the image plane. Both positive and negative areas of SHG-CD are observed in microscopy images, which relates to the anti-parallel arrangement of collagen fibres in different fascicles of the tendon. The theoretical formalism and experimental validation of DSMP imaging technique opens new opportunities for ultrastructural characterisation of chiral molecules, in particular collagen, and provides basis for the interpretation of SHG-CD signals. The nonlinear imaging of chiroptical parameters offers new possibilities to further improve the diagnostic sensitivity and/or specificity of nonlinear label-free histopathology.

## Introduction

Collagen is a major structural component of the extracellular matrix (ECM) in biological tissues and its structural organisation largely determines the mechanical and functional properties of tissues. For example, well-aligned collagen fibres provide the required viscoelastic properties of tendon and add elasticity to the composite material of bone. Further, the hierarchical structure of collagen determines differences in optical transparency of cornea and sclera^1–3^. In addition, structural alterations of collagen have been the focus of many studies related to ECM remodelling due to tumorigenesis^4–14^. Collagen alteration includes degradation of collagen in basement membranes^12^ and remodelling of fibrillar collagen (mainly collagen type-I) throughout the connective tissue of different organs^4,5,11,13,14^. Hence, detailed understanding of the structural properties of collagen is required to elucidate the details of collagen function and its role in disease-related processes.

One promising technique to visualise and quantify the structural properties of collagen assembly is second-harmonic generation (SHG) microscopy^15,16^. The efficiency of SHG generated by a sample is characterised by the second-order nonlinear susceptibility tensor, *χ*^(2)^, which contains information about the structure of the material and its spatial symmetry. Therefore, by extracting *χ*^(2)^ values, the organisation of collagen fibres can be characterised and certain molecular details about the collagen structure can be inferred. In addition, detailed knowledge of the *χ*^(2)^ tensor allows for better modelling of collagen molecular structure using ab initio calculations^17,18^. The ratio of independent components of the *χ*^(2)^ tensor has been extracted using various polarisation-resolved SHG (P-SHG) microscopy techniques^15,16,19–29^. In most studies, it is commonly assumed that the *χ*^(2)^ tensor components are real-valued when the fundamental and SHG signal frequencies are far from resonance frequencies in the material^8,24,29^. However, the *χ*^(2)^ tensor components are generally complex-valued and their imaginary parts can be related to the near-resonance enhancement of electric-dipole contributions and/or retardation of the nonlinear responses related to magnetic-dipole and electric-quadrupole effects^30–33^.

In order to extract both real and imaginary parts of the *χ*^(2)^ components, a recent technique called double Stokes-Mueller polarimetry (DSMP) can be used. The DSMP is a complete two-dimensional (2D) polarimetric SHG technique that is applicable for structural characterisation of optically-scattering samples^34,35^. In the DSMP formalism for SHG, the sample is represented by a 4 × 9 double Mueller matrix, which defines the relation between the polarisation states of incoming laser beam and the polarisation states of the outgoing SHG signal from the sample. Thirty-six measurements are needed to determine the Mueller matrix, from which the real and imaginary parts of six observable laboratory-frame *χ*^(2)^ tensor components can be extracted^36^. These components can further be used to extract the real and imaginary parts of the molecular susceptibility tensor component ratios. This is an extra step compared to our previous works where only real parts of susceptibilities were extracted^8,36^. The presence of imaginary components in *χ*^(2)^ has been related to chiroptical properties of collagen in thin films^31,37^.

Optical activity occurs in nonlinear optics when the nonlinear response of chiral molecules is different for right- and left-handed circularly polarised incoming laser light (RCP and LCP, respectively). The first demonstration of nonlinear optical activity was performed for SHG from chiral molecules deposited on surfaces^38,39^, in which the intensities of SHG for RCP and LCP were different. This response was considered as the equivalent of circular dichroism (CD) in linear optics. Hence, it is often referred to as SHG-CD. Since SHG is a surface-sensitive technique, its combination with CD provides an extremely sensitive tool for studying surface chirality. On the other hand, SHG-CD can also be useful in tissue imaging, such as for fast probing of nonlinear chiroptical properties of materials such as collagen, which is inherently chiral^40–43^. However, there are only a few studies on the origin of SHG-CD in biological tissues^44,45^ and, therefore, there is still a need to develop a model that relates the SHG-CD to the structural and organisational properties of collagen in tissue sections. Since the RCP and LCP incoming polarisation states are included in the DSMP formalism, the SHG-CD can be formulated using DSMP theory and can be deduced from a subset of a DSMP experiment. Using DSMP formalism and measurements, SHG-CD results can be related to the complex-valued chiral components of the susceptibility tensor and the 3D orientation of collagen fibres.

In this paper, DSMP is used to study the second-order nonlinear optical properties of collagen in thin tissue sections. Six observable complex-valued susceptibility tensor components are extracted. From these laboratory-frame components, three molecular-frame complex-valued susceptibility tensor component ratios are calculated for the first time. These reveal the presence of significant imaginary values of the chiral nonlinear susceptibility component, the phase and amplitude of which are also extracted. In addition, DSMP theory is used to formulate the SHG-CD response in terms of complex susceptibility tensor components and to determine the theoretical conditions under which SHG-CD has non-zero values. The SHG-CD response is deduced from a subset of DSMP measurements for a research model and the aforementioned theoretical conditions are experimentally validated.

## Methods

### Theoretical background

#### Double Stokes-Mueller polarimetry (DSMP) theory

For excitation objectives up to 0.8 NA, the polarization along the propagation axis is small and therefore a plane-wave approximation can be assumed^46^. A previous modelling showed that this assumption results in less than 10% over estimation of susceptibility components ratio^46^. Under a plane-wave approximation, the polarisation orientations of the focused incoming laser beam remain in the image plane, and a 2D polarimetry such as DSMP can be performed to extract six laboratory-frame *χ*^(2)^ tensor components^29^. In the DSMP formalism for SHG, the sample is represented by a 4 × 9 double Mueller matrix, ${{ {\mathcal M} }}^{(2)}$, whose elements are functions of the *χ*^(2)^ tensor components. A thin sample is assumed in this formalism (~5 μm) with negligible birefringence. For thicker samples, additional Mueller matrices can be introduced to account for the first order optical effects. The relation between the polarisation states of the incoming laser beam and the polarisation states of the outgoing SHG signal from the sample is defined using the nonlinear Stokes-Mueller polarimetry equation:

1 $$s^{\prime} (2\omega )={{ {\mathcal M} }}^{(2)}S(\omega )$$

where *s*′(2*ω*) is a 4 × 1 Stokes vector representing the outgoing SHG radiation and *S*(*ω*) is a 9 × 1 double Stokes vector representing the polarisation state of the incoming laser beam^34^. *S*(*ω*) consists of nine polarisation states as follows: horizontally (0°) and vertically (90°) linearly polarised (HLP and VLP, respectively), diagonally linearly polarised (±45°), RCP and LCP, linearly polarised at −22.5° and right- and left-hand elliptically polarised (REP and LEP, respectively). The Poincaré sphere coordinates of the states are given in previous publications^8,35^.

In a DSMP experiment, for each of the nine incoming polarisation states, four Stokes parameters of the outgoing SHG signal are recorded. To calculate the Mueller matrix elements, the scattering contribution from the dataset is removed by replacing the measured $\tilde{s}$ with calculated ${s}_{0}^{^{\prime} }=\sqrt{{\tilde{s}}_{1}^{2}+{\tilde{s}}_{2}^{2}+{\tilde{s}}_{3}^{2}}$ and keeping ${\tilde{s}}_{\gamma }={s}_{\gamma }^{^{\prime} }$ for *γ* = 1, 2, 3^36^. By having *S*(*ω*) from the prepared incoming states and $s\text{'}$ (2*ω*) from the measured SHG Stokes components, Eq. (1) can be used to obtain the Mueller matrix elements^34,35^. The double Mueller matrix depends on the sample structure and can be related to the second-order susceptibility tensor components, *χ*^(2)^, via *X*_rec_ matrix as follows^35^:

2 $${ {\mathcal M} }={T}{X}_{{\rm{rec}}}{{ {\mathcal H} }}^{-1}$$

where ${T}$ is a 4 × 4 matrix containing vectorized Pauli matrices as rows, and ${ {\mathcal H} }$ is a 9 × 9 matrix containing vectorized Gell-Mann matrices as rows^35^. The derivation and properties of these two matrices are described by Samim *et al*.^34^. They are both constant invertible matrices, which do not depend on the sample or the experimental setup and, hence, they only need to be calculated for SHG once. The *X*_rec_ matrix is the second-order susceptibility product matrix which contains products of susceptibility tensor components and their complex conjugates, and defined as^34^:

3 $${X}_{{\rm{rec}}}=\langle {\chi }^{(2)}\otimes {\chi }^{(2)\ast }\rangle =(\begin{array}{lllllllll}{\chi }_{11}{\chi }_{11}^{\ast } & {\chi }_{11}{\chi }_{12}^{\ast } & {\chi }_{11}{\chi }_{13}^{\ast } & {\chi }_{12}{\chi }_{11}^{\ast } & {\chi }_{12}{\chi }_{12}^{\ast } & {\chi }_{12}{\chi }_{13}^{\ast } & {\chi }_{13}{\chi }_{11}^{\ast } & {\chi }_{13}{\chi }_{12}^{\ast } & {\chi }_{13}{\chi }_{13}^{\ast }\\ {\chi }_{11}{\chi }_{21}^{\ast } & {\chi }_{11}{\chi }_{22}^{\ast } & {\chi }_{11}{\chi }_{23}^{\ast } & {\chi }_{12}{\chi }_{21}^{\ast } & {\chi }_{12}{\chi }_{22}^{\ast } & {\chi }_{12}{\chi }_{23}^{\ast } & {\chi }_{13}{\chi }_{21}^{\ast } & {\chi }_{13}{\chi }_{22}^{\ast } & {\chi }_{13}{\chi }_{23}^{\ast }\\ {\chi }_{21}{\chi }_{11}^{\ast } & {\chi }_{21}{\chi }_{12}^{\ast } & {\chi }_{21}{\chi }_{13}^{\ast } & {\chi }_{22}{\chi }_{11}^{\ast } & {\chi }_{22}{\chi }_{12}^{\ast } & {\chi }_{22}{\chi }_{13}^{\ast } & {\chi }_{23}{\chi }_{11}^{\ast } & {\chi }_{23}{\chi }_{12}^{\ast } & {\chi }_{23}{\chi }_{13}^{\ast }\\ {\chi }_{21}{\chi }_{21}^{\ast } & {\chi }_{21}{\chi }_{22}^{\ast } & {\chi }_{21}{\chi }_{23}^{\ast } & {\chi }_{22}{\chi }_{21}^{\ast } & {\chi }_{22}{\chi }_{22}^{\ast } & {\chi }_{22}{\chi }_{23}^{\ast } & {\chi }_{23}{\chi }_{21}^{\ast } & {\chi }_{23}{\chi }_{22}^{\ast } & {\chi }_{23}{\chi }_{23}^{\ast }\end{array})$$

where the indices, 1, 2, 3 are a contracted notation for the second-order susceptibility, ${\chi }_{iA}^{(2)}$ such that:

4 $$\begin{array}{llll}jk: & XX & ZZ & XZ,ZX\\ A: & 1 & 2 & 3\end{array}$$

In Eq. (3), an ensemble average is assumed for each element. The *X*_rec_ matrix can be obtained as:

5 $${X}_{{\rm{rec}}}={{T}}^{-1}{ {\mathcal M} }{ {\mathcal H} }$$

Hence, in order to recover an individual *χ*^(2)^ tensor component, the elements of *X*_rec_ have to be unmixed. First, *X*_rec_ is reshaped to a 6 × 6 square coherency matrix, *X*:

6 $$X=(\begin{array}{llll}{\chi }_{11}{\chi }_{11}^{\ast } & {\chi }_{11}{\chi }_{12}^{\ast } & \mathrm{...} & {\chi }_{11}{\chi }_{23}^{\ast }\\ {\chi }_{12}{\chi }_{11}^{\ast } & {\chi }_{12}{\chi }_{12}^{\ast } &  & {\chi }_{12}{\chi }_{23}^{\ast }\\ \mathrm{...} &  &  & \\ {\chi }_{23}{\chi }_{11}^{\ast } & {\chi }_{23}{\chi }_{12}^{\ast } & \mathrm{...} & {\chi }_{23}{\chi }_{23}^{\ast }\end{array})$$

It can be assumed that one of the *X* elements (e.g. *χ*_11_) is real and positive, so that

7 $${X}_{11}={\chi }_{11}{\chi }_{11}^{\ast }={\chi }_{11}^{2}\Rightarrow {\chi }_{11}=\sqrt{{X}_{11}}$$

where the positive sign is taken by definition. The remaining components can be obtained from the off-diagonal elements involving the known positive real-valued *χ* (*χ*_11_ in this case) as follows:

8 $${\chi }_{12}=\frac{{X}_{21}}{{\chi }_{11}},{\chi }_{13}=\frac{{X}_{31}}{{\chi }_{11}},\,\ldots $$

Note that the amplitude and sign of the other elements are relative to *χ*_11_. The off-diagonal elements can be complex-valued, indicating that the *χ* values can have a retardance with respect to each other. By taking the real and imaginary parts, the complex value of each observable *χ* component can be deduced. Consequently, the amplitudes of each susceptibility component as well as their relative phase differences can be calculated.

#### Orientation dependent second-order susceptibility of collagen

The measurement of the *χ*^(2)^ tensor components is performed in the laboratory coordinate system, as indicated by the uppercase *IJK*/*XYZ* indices. In this coordinate system, the sample is placed in the *XZ*-plane, the laser beam propagates along the *Y*-axis and the second-order nonlinear susceptibility tensor is indicated as ${\chi }_{ijk}^{(2)}$. As it mentioned, in 2D polarimetry where the polarisation orientations of the focused incoming laser beam remain in the *XZ* image plane, only six laboratory-frame *χ*^(2)^ tensor components can be measured^29^: ${\chi }_{XXX}^{(2)}$, ${\chi }_{XXZ}^{(2)}$, ${\chi }_{XZZ}^{(2)}$, ${\chi }_{ZXX}^{(2)}$, ${\chi }_{ZZX}^{(2)}$, ${\chi }_{ZZZ}^{(2)}$, where ${\chi }_{XXZ}^{(2)}$ = ${\chi }_{XZX}^{(2)}$ and ${\chi }_{ZZX}^{(2)}$ = ${\chi }_{ZXZ}^{(2)}$ for the SHG process. The *χ*^(2)^ tensor components in the laboratory coordinate system can be expressed in terms of the molecular second-order susceptibility tensor components, ${\chi }_{ijk}^{(2)}$, and the 3D orientation of the sample with respect to the laboratory frame of reference as follows^29^:

9a $$\begin{array}{ccc}{\chi }_{XXX}^{(2)} & = & \cos \,\alpha \,\sin \,\delta [({\chi }_{zzz}^{(2)}-2{\chi }_{xxz}^{(2)}-{\chi }_{zxx}^{(2)}){\cos }^{2}\alpha \,{\sin }^{2}\delta +2{\chi }_{xxz}^{(2)}+{\chi }_{zxx}^{(2)}]\end{array}$$

9b $$\begin{array}{ccc}{\chi }_{XXZ}^{(2)} & = & \cos \,\alpha \,\cos \,\delta [({\chi }_{zzz}^{(2)}-2{\chi }_{xxz}^{(2)}-{\chi }_{zxx}^{(2)}){\cos }^{2}\alpha \,{\sin }^{2}\delta +{\chi }_{xxz}^{(2)}]-\frac{1}{2}{\chi }_{xyz}^{(2)}\,\sin (2\alpha )\,\sin \,\delta \end{array}$$

9c $$\begin{array}{ccc}{\chi }_{XZZ}^{(2)} & = & \cos \,\alpha \,\sin \,\delta [({\chi }_{zzz}^{(2)}-2{\chi }_{xxz}^{(2)}-{\chi }_{zxx}^{(2)}){\cos }^{2}\alpha \,{\cos }^{2}\delta +{\chi }_{zxx}^{(2)}]-{\chi }_{xyz}^{(2)}\,\sin \,2\alpha \,\cos \,\delta \end{array}$$

9d $$\begin{array}{ccc}{\chi }_{ZXX}^{(2)} & = & \cos \,\alpha \,\cos \,\delta [({\chi }_{zzz}^{(2)}-2{\chi }_{xxz}^{(2)}-{\chi }_{zxx}^{(2)}){\cos }^{2}\alpha \,{\sin }^{2}\delta +{\chi }_{zxx}^{(2)}]+{\chi }_{xyz}^{(2)}\,\sin (2\alpha )\,\sin \,\delta \end{array}$$

9e $$\begin{array}{ccc}{\chi }_{ZXZ}^{(2)} & = & \cos \,\alpha \,\sin \,\delta [({\chi }_{zzz}^{(2)}-2{\chi }_{xxz}^{(2)}-{\chi }_{zxx}^{(2)}){\cos }^{2}\alpha \,{\cos }^{2}\delta +{\chi }_{xxz}^{(2)}]+\frac{1}{2}{\chi }_{xyz}^{(2)}\,\sin (2\alpha )\,\cos \,\delta \end{array}$$

9f $$\begin{array}{ccc}{\chi }_{ZZZ}^{(2)} & = & \cos \,\alpha \,\cos \,\delta [({\chi }_{zzz}^{(2)}-2{\chi }_{xxz}^{(2)}-{\chi }_{zxx}^{(2)}){\cos }^{2}\alpha \,{\cos }^{2}\delta +2{\chi }_{xxz}^{(2)}+{\chi }_{zxx}^{(2)}]\end{array}$$

where *α* is the out-of-plane tilt angle with respect to the *XZ* image plane, and *δ* is the average in-plane fibre orientation angle measured from the Z-axis in the laboratory frame of reference. The terms containing ${\chi }_{xyz}^{(2)}$ tensor components represent the chiral properties^29^. Equations (9) indicate that the susceptibility tensor components in laboratory coordinate system can be written as an achiral contribution plus a chiral contribution that depends on ${\chi }_{xyz}^{(2)}$ and *α*. Consequently, for the collagen fibres that are in the image plane (*α* = 0) the contribution of the chiral terms is zero, so that the tensor is indistinguishable between a chiral and an achiral structure.

#### SHG circular dichroism (SHG-CD)

SHG-CD is defined as the normalised difference in outgoing intensity of SHG when the sample is excited with RCP and LCP light, (*I*_RCP_−*I*_LCP_)/((*I*_RCP_ + *I*_LCP_)/2). Since two of the nine incoming polarisation states are RCP and LCP, DSMP data can be used to extract the SHG-CD of the sample. The SHG-CD measured using DSMP technique can be written in terms of the Stokes vector parameters, which in turn can be expressed in terms of Mueller matrix elements by using Eq. (1) and substituting the double Stokes vector parameters^34^:

10 $${\rm{SHG}} \mbox{-} {\rm{CD}}=2\frac{{s}_{0,{\rm{RCP}}}^{^{\prime} }-{s}_{0,{\rm{LCP}}}^{^{\prime} }}{{s}_{0,{\rm{RCP}}}^{^{\prime} }+{s}_{0,{\rm{LCP}}}^{^{\prime} }}=2\frac{{s}_{0,5}^{^{\prime} }-{s}_{0,6}^{^{\prime} }}{{s}_{0,5}^{^{\prime} }+{s}_{0,6}^{^{\prime} }}=4\frac{{{ {\mathcal M} }}_{0,8}-{{ {\mathcal M} }}_{0,9}}{\sqrt{6}{{ {\mathcal M} }}_{0,1}-\sqrt{3}{{ {\mathcal M} }}_{0,2}-{{ {\mathcal M} }}_{0,4}}$$

The Mueller matrix elements were shown to be functions of the products of the second-order susceptibility tensor components in the laboratory coordinate system, ${\chi }_{IJK}^{(2)}$, as follows^34^:

11a $${{ {\mathcal M} }}_{0,1}=\frac{\sqrt{6}}{6}((|{\chi }_{ZXX}^{(2)}{|}^{2}+|{\chi }_{ZZZ}^{(2)}{|}^{2}+|{\chi }_{ZXZ}^{(2)}{|}^{2})+(|{\chi }_{XXX}^{(2)}{|}^{2}+|{\chi }_{XZZ}^{(2)}{|}^{2}+|{\chi }_{XXZ}^{(2)}{|}^{2}))$$

11b $${{ {\mathcal M} }}_{0,2}=\frac{\sqrt{3}}{6}((|{\chi }_{ZXX}^{(2)}{|}^{2}+|{\chi }_{ZZZ}^{(2)}{|}^{2}-2|{\chi }_{ZXZ}^{(2)}{|}^{2})+(|{\chi }_{XXX}^{(2)}{|}^{2}+|{\chi }_{XZZ}^{(2)}{|}^{2}-2|{\chi }_{XXZ}^{(2)}{|}^{2}))$$

11c $${{ {\mathcal M} }}_{0,4}=|{\chi }_{ZZZ}^{(2)}||{\chi }_{ZXX}^{(2)}|{\cos {\rm{\Delta }}}_{ZZZ,ZXX}+|{\chi }_{XXX}^{(2)}||{\chi }_{XZZ}^{(2)}|{\cos {\rm{\Delta }}}_{XXX,XZZ}$$

11d $${{ {\mathcal M} }}_{0,8}=|{\chi }_{ZZZ}^{(2)}||{\chi }_{ZXZ}^{(2)}|{\sin {\rm{\Delta }}}_{ZZZ,ZXZ}+|{\chi }_{XZZ}^{(2)}||{\chi }_{XXZ}^{(2)}|{\sin {\rm{\Delta }}}_{XZZ,XXZ}$$

11e $${{ {\mathcal M} }}_{0,9}=|{\chi }_{ZXX}^{(2)}||{\chi }_{ZXZ}^{(2)}|{\sin {\rm{\Delta }}}_{ZXX,ZXZ}+|{\chi }_{XXX}^{(2)}||{\chi }_{XXZ}^{(2)}|{\sin {\rm{\Delta }}}_{XXX,XXZ}$$

Here, ${\chi }_{IJK}^{(2)}$ is assumed to be complex and is written as ${\chi }_{IJK}^{(2)}=|{\chi }_{IJK}^{(2)}|{e}^{i{\xi }_{IJK}}$. Therefore, ${{\rm{\Delta }}}_{IJK,I\text{'}J\text{'}K\text{'}}={\xi }_{IJK}-{\xi }_{I\text{'}J\text{'}K\text{'}}$ is the associated phase difference between different laboratory frame susceptibility tensor components. By substituting Eqs (9) and (11) in Eq. (10), the SHG-CD can be obtained in terms of the products of the magnitudes of the molecular susceptibility tensor components, $|{\chi }_{ijk}^{(2)}||{\chi }_{i\text{'}j\text{'}k\text{'}}^{(2)}|$, the associated molecular phase differences, ${{\rm{\Delta }}}_{ijk,i\text{'}j\text{'}k\text{'}}={{\zeta }}_{ijk}-{{\zeta }}_{i\text{'}j\text{'}k\text{'}}$, and the tilt angle of collagen fibres out of the image plane. Similar to ${\chi }_{IJK}^{(2)}$, ${\chi }_{ijk}^{(2)}$ is assumed to be complex and is written as ${\chi }_{ijk}^{(2)}=|{\chi }_{ijk}^{(2)}|{e}^{i{\zeta }_{ijk}}$. The numerator and denominator of Eq. (10) can be expressed as

12 $${\rm{SHG}} \mbox{-} {{\rm{CD}}}_{{\rm{numerator}}}=2|{\chi }_{xyz}^{(2)}|\sin (2\alpha ){\cos }^{3}(\alpha )[|{\chi }_{zzz}^{(2)}|{\sin {\rm{\Delta }}}_{zzz,xyz}-|{\chi }_{zxx}^{(2)}|{\sin {\rm{\Delta }}}_{zxx,xyz}-2|{\chi }_{xxz}^{(2)}|{\sin {\rm{\Delta }}}_{xxz,xyz}]$$

13 $$\begin{array}{rcl}{\rm{SHG}} \mbox{-} {{\rm{CD}}}_{{\rm{denominator}}} & = & 0.5{|{\chi }_{zzz}^{(2)}|}^{2}{\cos }^{6}(\alpha )+0.5{|{\chi }_{zxx}^{(2)}|}^{2}{\cos }^{6}(\alpha )\\  &  & +\,2{|{\chi }_{xxz}^{(2)}|}^{2}({\cos }^{6}\alpha -2{\cos }^{4}\alpha +2{\cos }^{2}\alpha )\\  &  & -|{\chi }_{zzz}^{(2)}||{\chi }_{zxx}^{(2)}|{\cos {\rm{\Delta }}}_{zzz,zxx}{\cos }^{6}\alpha \\  &  & +\,2|{\chi }_{zzz}^{(2)}||{\chi }_{xxz}^{(2)}|{\cos {\rm{\Delta }}}_{zzz,xxz}({\cos }^{4}\alpha -{\cos }^{6}\alpha )\\  &  & +\,2|{\chi }_{zxx}^{(2)}||{\chi }_{xxz}^{(2)}|{\cos {\rm{\Delta }}}_{zxx,xxz}({\cos }^{6}\alpha -{\cos }^{4}\alpha )+{|{\chi }_{xyz}^{(2)}|}^{2}{\sin }^{2}2\alpha \end{array}$$

The in-plane fibre angle, *δ*, is absent from Eqs (12) and (13) indicating that, when the incoming polarisation is circular, SHG-CD is independent of the orientation of the sample in the image plane. Using Eq. (13), it can be concluded that three conditions have to be fulfilled simultaneously in order to observe SHG-CD effects: (i) the sample (collagen fibres in this case) has to be tilted out of the image plane (i. e. *α* ≠ 0), (ii) the sample has to be chiral (${\chi }_{xyz}^{(2)}\ne 0$), and (iii) there has to be a phase difference between the chiral term, ${\chi }_{xyz}^{(2)}$, and achiral terms, ${\chi }_{zzz}^{(2)}$, ${\chi }_{zxx}^{(2)}$ or ${\chi }_{xxz}^{(2)}$ (i.e. ${{\rm{\Delta }}}_{ijk,i\text{'}j\text{'}k\text{'}}$≠ 0). These conditions are very similar to those reported for non-vanishing surface SHG-CD^33^.

According to Eqs (12) and (13), the SHG-CD depends on the sign and magnitudes of the chiral term as well as on the sign of the angle *α*. It is interesting to note that Eq. (12) predicts that, when the fibre orientation flips, the sign of the SHG-CD changes. This can be understood qualitatively by rotating the collagen fibre by *α* = 180°. In this situation, *x*→*x*, *y* → −*y*, *z* → −*z*. Hence, the tensor components ${\chi }_{zzz}^{(2)}$, ${\chi }_{zxx}^{(2)}$ or ${\chi }_{xxz}^{(2)}$ change their sign, while the sign of ${\chi }_{xyz}^{(2)}$ does not change.

The SHG-CD also depends on the interaction of the chiral and achiral terms through ${{\rm{\Delta }}}_{ijk,i\text{'}j\text{'}k\text{'}}$. Within the electric-dipole approximation, this phase difference may exist if the fundamental and/or second-harmonic frequencies are close to resonance frequencies of the material. On the other hand, when magnetic-dipole and electric-quadrupole interactions are included, the required phase difference will exist also for non-resonant conditions. The SHG-CD can also approach zero for certain ${\chi }_{zzz}^{(2)}$, ${\chi }_{zxx}^{(2)}$, and ${\chi }_{xxz}^{(2)}$ and corresponding ${{\rm{\Delta }}}_{ijk,i\text{'}j\text{'}k\text{'}}$ values constituting the expression in the brackets of Eq. (12). This effect may become noticeable when ${\chi }_{zzz}^{(2)}/{\chi }_{zxx}^{(2)} \sim 3$ and ${\chi }_{xxz}^{(2)}/{\chi }_{zxx}^{(2)} \sim 1$.

### DSMP microscope

A custom-built diode-pumped ytterbium-ion-doped potassium gadolinium tungstate (Yb:KGW) crystal-based oscillator was used as the laser source. It operates at 1028 nm and it generates ~400 fs pulses at 14.3 MHz repetition rate^47^. The laser was coupled to a custom-built laser-scanning SHG microscope, as described previously^36^. A 20 × 0.75 numerical aperture (NA) air objective (Carl Zeiss, Thornwood, New York, USA) was used to focus the beam onto the sample, and a custom 0.85 NA objective (Omex Technologies, Wheeling, Illinois, USA) was used to collect the SHG signal in the transmission geometry. The SHG signal was detected using a single-photon-counting photomultiplier tube (Hamamatsu H10682-210, Hamamatsu, Bridgewater, New Jersey, USA). A BG 39 filter and a 510–520 nm band-pass interference filter (Edmund Optics, Regina, Saskatchewan, Canada) were used to separate the SHG from the incident light. The microscope polarimeter consisted of a polarisation-state generator (PSG) inserted before the excitation objective and a polarisation-state analyser (PSA) placed after the collection objective. The PSG consisted of a fixed linear polariser (Thorlabs, Newton, New Jersey, USA), a half-waveplate (HWP) and a quarter-waveplate (QWP). The PSA contains a HWP, a QWP (all wave plates are from Eksma Optics, Lithuania) and a linear polarizer (Thorlabs, Newton, New Jersey, USA). The PSG was set to produce nine different incoming polarisation states (HLP, VLP, +45, −45, RCP, LCP, −22.5, REP, LEP), for each of which the PSA was set to measure the SHG signal at six different polarisation states (0, 90, +45, −45, RCP, LCP) in order to determine the SHG Stokes vector.

### Sample preparation

Collagen type-I from Achilles tendon of a 25 kg Yorkshire pig was studied. Pig tendon was chosen because it is a well-ordered structure and due to its size, it can be easily cut at various angles. The tendon was harvested from a healthy dead pig euthanized after an unrelated study with approval of the Animal Care Committee of the University Health Network, Toronto, Canada. The tendon was formalin fixed and cut along the tendon axis (longitudinal cut), at *α* = 30° (oblique cut) and perpendicular to the tendon axis (transverse cut). The samples were embedded in paraffin and cut into 5-μm thick sections. The sections were mounted on glass slides and stained with hematoxylin and eosin (H&E) to provide anatomical reference recorded with bright-field whole-slide scanner (Aperio: Leica Biosystems). One slide per cut angle was studied.

## Results and Discussion

### Extraction of complex *χ*^(2)^ using DSMP microscopy

**Figure 1:**
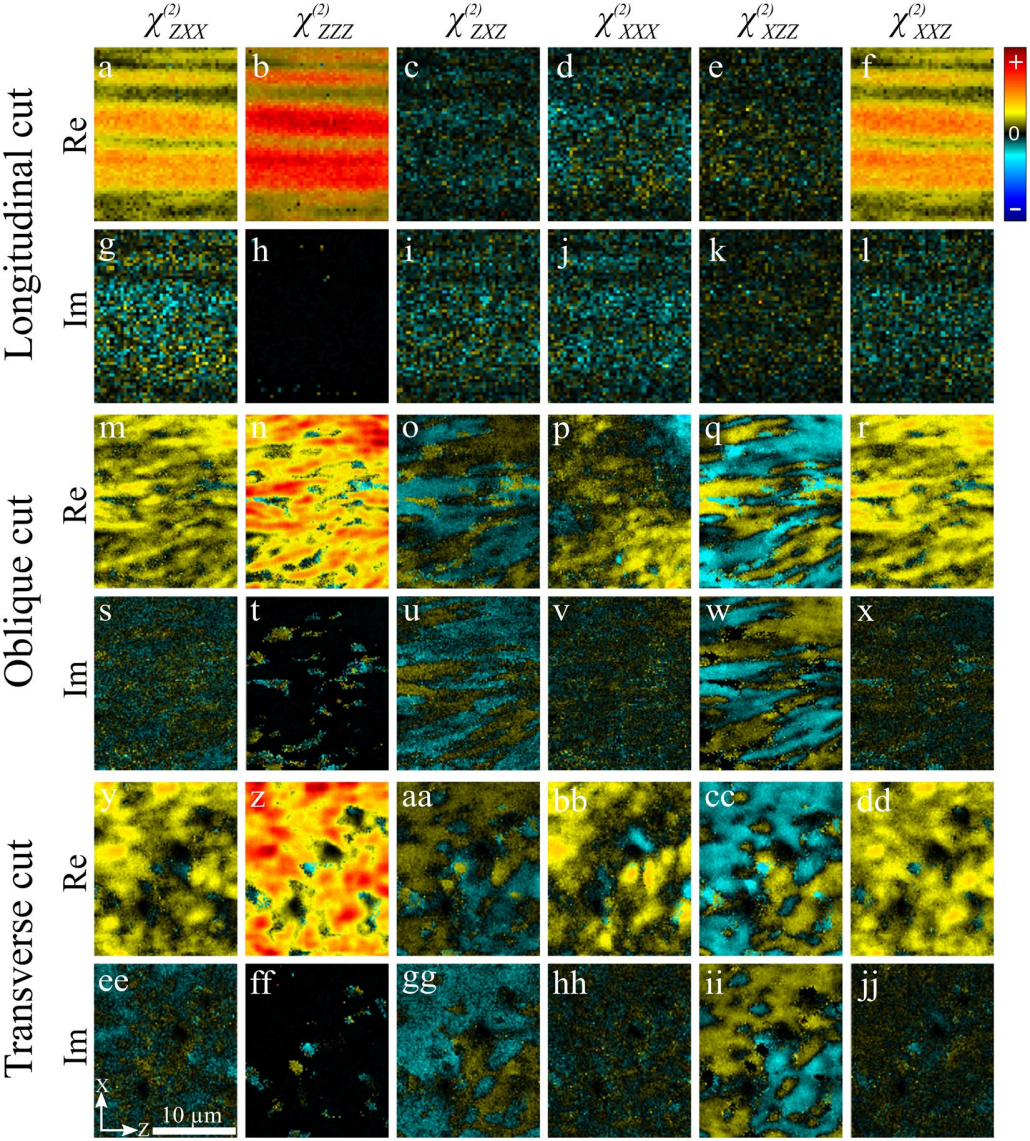
Measured real and imaginary parts of the six observable laboratory-frame nonlinear susceptibility *χ*^(2)^ tensor components in pig tendon cut longitudinally, obliquely and transversely. The laboratory coordinates defining the image plane (*XZ*) and the scale bar are shown in panel ee. The colour bar is shown in upper right corner.

DSMP microscopy was used to extract the real and imaginary parts of the six observable susceptibility tensor components for each of the three cut angles in the tendon: Fig. 1. All the components were normalised to ${\chi }_{ZZZ}^{(2)}$ which was assumed to be real-valued and positive. For the longitudinal cut the collagen fibres are closely aligned with the laboratory frame of reference (*δ*~0 and *α*~0) and it can be seen that the real part of ${\chi }_{ZZZ}^{(2)}$ (Fig. 1b) is maximum, as expected from Eq. (9f). In addition, the real parts of ${\chi }_{XXZ}^{(2)}$ and ${\chi }_{ZXX}^{(2)}$ (Fig. 1a,f) are similar to each other, which is in agreement with Eq. (9b,d) when *δ*~0 and *α*~0. The real values of the ${\chi }_{ZXZ}^{(2)}$ and ${\chi }_{XZZ}^{(2)}$ components are as small as that of the ${\chi }_{XXX}^{(2)}$ component (Fig. 1c–e, respectively), and can be considered negligible. This observation is also in agreement with Eq. (9c,e) when *δ*~0 and *α*~0. The imaginary parts of all susceptibility components are small at this cut angle, indicating that all the observable susceptibility components are in phase with each other and so real-valued.

For the obliquely-cut sample, all real susceptibility components have significant values, with the largest occurring in ${\chi }_{ZZZ}^{(2)}$ (Fig. 1n) and with much smaller values in ${\chi }_{XXX}^{(2)}$ (Fig. 1p), indicating that projections of collagen fibres onto the image plane are predominantly, but not perfectly, aligned with the laboratory *Z*-axis, meaning that *δ*~0. This can also explain why the real parts of ${\chi }_{ZXX}^{(2)}$ and ${\chi }_{XXZ}^{(2)}$ have the same signs (Fig. 1m,r, respectively), while ${\chi }_{ZXZ}^{(2)}$ and ${\chi }_{XZZ}^{(2)}$ (Fig. 1o,q, respectively) have anti-correlated positive and negative values. For the ${\chi }_{ZXX}^{(2)}$ and ${\chi }_{XXZ}^{(2)}$ components the chiral contribution scales as sin *δ*, while the achiral contribution scales as cos *δ* (see Eq. (9b,d)). Therefore, for fibres that are aligned with the laboratory *Z*-axis (*δ*~0), the achiral terms are larger than the chiral terms, which results in the same sign for ${\chi }_{ZXX}^{(2)}$ and ${\chi }_{XXZ}^{(2)}$. In contrast, for the ${\chi }_{ZXZ}^{(2)}$ and ${\chi }_{XZZ}^{(2)}$ components, the chiral contribution scale as cos *δ*, while achiral contributions scale as sin *δ*, so that the chiral contribution is larger than the achiral contribution, resulting in opposite signs for fibres oriented close to *δ* = 0. Further, large and anti-correlated imaginary contributions are observed for ${\chi }_{ZXZ}^{(2)}$ and ${\chi }_{XZZ}^{(2)}$ (Fig. 1u,w, respectively), pointing to a significant imaginary ${\chi }_{xyz}^{(2)}$ contribution.

For the transverse cut, the collagen fibres are closer to *α* = 90°, so that the SHG signal decreases due to symmetry considerations, resulting in a lower signal-to-noise ratio (SNR). Hence, fibres deviating from *α* = 90° show larger signal, so that care should be taken when interpreting the results. The transverse cut shows similar properties to the oblique cut, with emerging islands of anti-correlated positive and negative areas in ${\chi }_{XZZ}^{(2)}$ and ${\chi }_{ZXZ}^{(2)}$ for both the real and imaginary parts (Fig. 1aa,cc,gg,ii, respectively). The ${\chi }_{XXZ}^{(2)}$ and ${\chi }_{ZXX}^{(2)}$ images correlate, having the same sign, similar to the oblique cut (Fig. 1y,dd).

**Figure 2:**
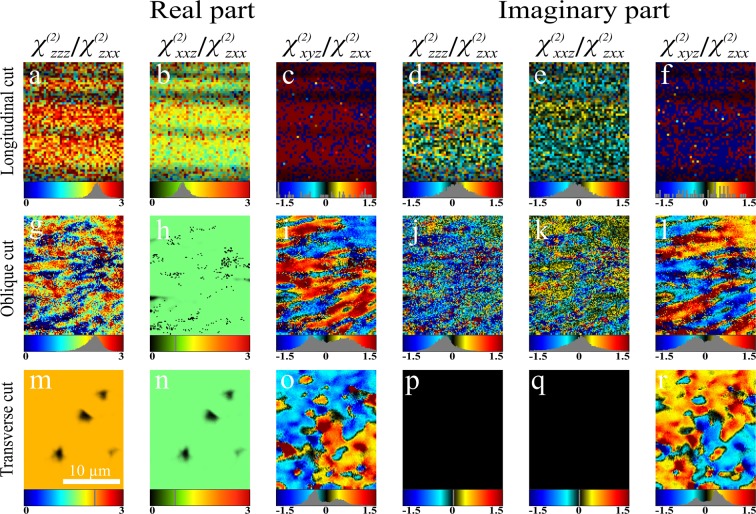
The real and imaginary parts of the relative molecular susceptibility components, ${\chi }_{zzz}^{(2)}/{\chi }_{zxx}^{(2)}$, ${\chi }_{xxz}^{(2)}/{\chi }_{zxx}^{(2)}$, and ${\chi }_{xyz}^{(2)}/{\chi }_{zxx}^{(2)}$ extracted from the measured laboratory-frame nonlinear susceptibility tensor components. The ratio value distribution and colour representation are shown at the bottom of each panel. The scale bar is indicated in panel m.

The six observable susceptibility tensor components in the laboratory frame can be fitted by Eq. (9) to extract the real and imaginary parts of the relative molecular susceptibility components, ${\chi }_{zzz}^{(2)}/{\chi }_{zxx}^{(2)}$, ${\chi }_{xxz}^{(2)}/{\chi }_{zxx}^{(2)}$ and ${\chi }_{xyz}^{(2)}/{\chi }_{zxx}^{(2)}$. In order to reduce the number of fitting parameters, the *α* values were estimated using only linear polarisation measurements, as previously described^29^. These values are: 0° for longitudinal cut, 30° for the oblique cut and 70° for the transverse cut. The fitting results are shown in Fig. 2. For the longitudinal cut, the real part of ${\chi }_{zzz}^{(2)}/{\chi }_{zxx}^{(2)}$ (Re(${\chi }_{zzz}^{(2)}/{\chi }_{zxx}^{(2)}$)), has a normal distribution with a mean value of 1.73 ± 0.01 and a full width at half maximum (FWHM) of 0.73 ± 0.03 (Fig. 2a). This mean value is in agreement with the previously reported 1.67 ± 0.05 for pig tendon^29^. Re(${\chi }_{xxz}^{(2)}/{\chi }_{zxx}^{(2)}$) also is normally distributed with a mean value of 1.08 ± 0.01 and FWHM of 0.44 ± 0.03 (Fig. 2b), indicating that Re(${\chi }_{xxz}^{(2)}/{\chi }_{zxx}^{(2)}$) = 1 is a valid assumption. The Re(${\chi }_{xyz}^{(2)}/{\chi }_{zxx}^{(2)}$) values are dispersed and can be considered to be zero, indicating that no chiral contribution can be observed for collagen fibres lying in the image plane (Fig. 2c).

The imaginary values of ${\chi }_{zzz}^{(2)}/{\chi }_{zxx}^{(2)}$ and ${\chi }_{xxz}^{(2)}/{\chi }_{zxx}^{(2)}$ are distributed normally around 0.06 ± 0.01 and −0.03 ± 0.01, respectively (Fig. 2d,e). Similar to the Re(${\chi }_{xyz}^{(2)}/{\chi }_{zxx}^{(2)}$) distribution, Im(${\chi }_{xyz}^{(2)}/{\chi }_{zxx}^{(2)}$) values are dispersed and can be considered to be zero (Fig. 2f). Thus, for longitudinal cuts, there are no significant imaginary components for any of the molecular susceptibility ratios.

In order to perform the fitting on the oblique cut, the real part of ${\chi }_{xxz}^{(2)}/{\chi }_{zxx}^{(2)}$ was fixed at 1 to decrease the number of free parameters. As seen in Fig. 2g, Re(${\chi }_{zzz}^{(2)}/{\chi }_{zxx}^{(2)}$) is normally distributed around 1.68 ± 0.02, which is not statistically significantly different from the value for the longitudinal cut: the slight difference could be due to fixing Re(${\chi }_{xxz}^{(2)}/{\chi }_{zxx}^{(2)}$) to unity. The Re(${\chi }_{xxz}^{(2)}/{\chi }_{zxx}^{(2)}$) is all in green because this parameter was fixed to 1 during the fitting (Fig. 2h). In contrast to the longitudinal cut, Re(${\chi }_{xyz}^{(2)}/{\chi }_{zxx}^{(2)}$) shows non-zero values (Fig. 2i), with both positive and negative values being present and showing a binomial distribution of the chiral ratio. A double-Gaussian was fitted to this distribution and a weighted average of the module of the two means was calculated, resulting in Re(${\chi }_{xyz}^{(2)}/{\chi }_{zxx}^{(2)}$) = 0.39 ± 0.05. This value is similar to the previously reported chiral ratio of 0.29±0.04 for collagen in pig tendon^29^. The mean values of the normally distributed Im(${\chi }_{zzz}^{(2)}/{\chi }_{zxx}^{(2)}$) and Im(${\chi }_{xxz}^{(2)}/{\chi }_{zxx}^{(2)}$) are −0.1 ± 0.03 and 0.01 ± 0.03, respectively (Fig. 2j,k), which are not statistically significantly different from zero, indicating that imaginary parts of achiral components ${\chi }_{zzz}^{(2)}/{\chi }_{zxx}^{(2)}$ and ${\chi }_{xxz}^{(2)}/{\chi }_{zxx}^{(2)}$ are not influenced by the tilt angle *α* and the ${\chi }_{zzz}^{(2)}$, ${\chi }_{zxx}^{(2)}$ and ${\chi }_{xxz}^{(2)}$ susceptibilities are in phase with respect to each other. Im(${\chi }_{xyz}^{(2)}/{\chi }_{zxx}^{(2)}$) on the other hand shows pronounced values both in the positive and negative ranges (Fig. 2l), indicating that the ${\chi }_{xyz}^{(2)}$ component has a phase shift with respect to ${\chi }_{zzz}^{(2)}$, ${\chi }_{zxx}^{(2)}$ and ${\chi }_{xxz}^{(2)}$. Similar to the real part, a double-Gaussian was fitted to the Im(${\chi }_{xyz}^{(2)}/{\chi }_{zxx}^{(2)}$) distribution and yielded a weighted average of 0.31 ± 0.05.

The results from the longitudinal and oblique cuts showed that the real part of ${\chi }_{zzz}^{(2)}/{\chi }_{zxx}^{(2)}$ remains constant, as expected since this is structural property of the fibres and so should not depend on their 3D orientation. In addition, the results suggest that the imaginary parts of the ${\chi }_{zzz}^{(2)}/{\chi }_{zxx}^{(2)}$ and ${\chi }_{xxz}^{(2)}/{\chi }_{zxx}^{(2)}$ are negligible and independent of *α*. Hence, to reduce the number of fitting parameters for the transverse cut, the fitting was performed by fixing Re(${\chi }_{zzz}^{(2)}/{\chi }_{zxx}^{(2)}$) = 1.7, Im(${\chi }_{zzz}^{(2)}/{\chi }_{zxx}^{(2)}$) = 0, Re(${\chi }_{xxz}^{(2)}/{\chi }_{zxx}^{(2)}$) = 1 and Im(${\chi }_{xxz}^{(2)}/{\chi }_{zxx}^{(2)}$)=0. Under these constrains, the real and imaginary parts of the chiral contribution were fitted, resulting in binomial distributions (Fig. 2o,r, respectively). The weighted average of the modulus of Re(${\chi }_{xyz}^{(2)}/{\chi }_{zxx}^{(2)}$) was 0.37 ± 0.04. Similar to the oblique cut, Im(${\chi }_{xyz}^{(2)}/{\chi }_{zxx}^{(2)}$) of the transverse cut showed a binomial distribution with a weighted average of the modulus of 0.34 ± 0.05.

The observations demonstrate that, although the second-order susceptibility components of collagen fibres have a chiral contribution, they can only be observed when the fibres are tilted out of the image plane (*α* ≠ 0). The presence of imaginary values only for the chiral susceptibility suggests a phase retardation between chiral (${\chi }_{xyz}^{(2)}$) and achiral (${\chi }_{zzz}^{(2)}$, ${\chi }_{zxx}^{(2)}$ and ${\chi }_{xxz}^{(2)}$) components. Previous studies have used a complex ${\chi }_{xyz}^{(2)}$ component to model SHG-CD, which resulted in qualitative agreement between SHG-CD simulations and experiments^44^. The phase difference can occur due to presence of a resonance transition near either the fundamental laser wavelengths and/or the SHG wavelengths. The wavelength that was used in this study however, was far from the collagen absorption wavelength which was shown to be around 200 nm^48^. Complex-valued susceptibilities can also appear due to the contribution of magnetic dipoles in the nonlinear optical interaction. In this situation, off-resonance complex-valued susceptibilities can be observed. In addition, the electric quadrupole effects can lead to phase retardation between the susceptibility components^30,39,49^. Performing a spectroscopic DSMP measurement will shine light to the actual origin of the imaginary components of *χ*^(2)^ and consequently the chiroptical properties of collagen.

**Figure 3:**
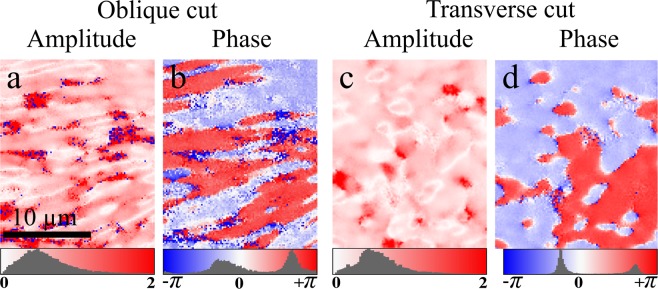
Amplitude (**a**,**c**) and phase (**b**,**d**) of the chiral ratio in samples with oblique and transverse cuts. The occurrence histograms and colour bars are shown below each image.

Given the real and imaginary parts of the chiral susceptibility component ratio, the phase and amplitude of the chiral ratio for each pixel can be extracted. Figure 3 shows colour maps of the amplitudes (Fig. 3a,c) and phases (Fig. 3b,d) of ${\chi }_{xyz}^{(2)}/{\chi }_{zxx}^{(2)}$ for each pixel for the different cut angles. The occurrence histogram of the amplitudes and the phases are shown below each image. The amplitude of the chiral ratio shows a small variation centred around $|{\chi }_{xyz}^{(2)}/{\chi }_{zxx}^{(2)}|=0.65\pm 0.02$ for the oblique cut and 0.52 ± 0.01 for the transverse cut. The phase varies from −*π* to *π* and the corresponding values are represented from blue to red. The occurrence histograms of the phase show two peaks occurring at −44 ± 1° and 122 ± 1° for the oblique cut and −46 ± 1° and 128 ± 1° for the transverse cut. Thus, the differences between the two peaks are 166 ± 3° and 174 ± 1°, respectively. This can be attributed to the anti-parallel arrangement of collagen fibres in the tendon fascicle, as previously reported^50–54^.

The ${\chi }_{xyz}^{(2)}/{\chi }_{zxx}^{(2)}$ amplitude and phase are the two new parameters extracted for the first time. The amplitude can be used to detect variation in collagen absolute chirality over the image. Figure 3a,c show that most of the fascicles in pig tendon have similar chirality amplitude albeit opposite polarity, while only a few areas show a slightly different amplitude. It can also be seen that the borders between opposite polarities show slightly lower chiral ratio amplitude. It is likely that collagen with different chirality amplitude can be revealed in the amplitude image, and could present an interesting avenue to explore the possibility of separating different types of collagen in the same tissue. On the other hand, the sign of phase image is sensitive to the polarity of collagen (Fig. 3b,d). Therefore, phase image can be used to investigate polar arrangement of collagen in the tissue at the diffraction-limited resolution.

### SHG-CD of collagen

**Figure 4:**
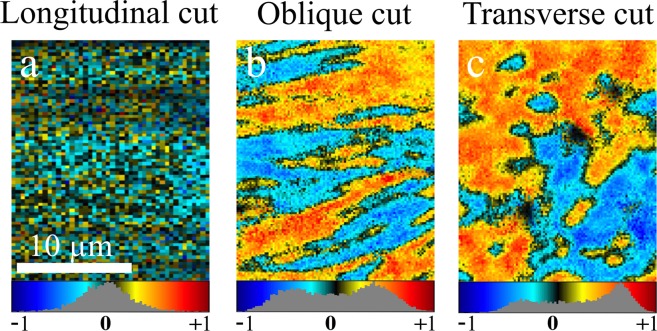
SHG-CD images of tendon at different cut angles: (**a**) longitudinal, (**b**) oblique, (**c**) transverse. The occurrence histograms and colour bars are shown below each image.

The data for SHG-CD involving RCP and LCP incoming polarisations are contained in the full DSMP measurement, so that the calculated parameters from DSMP presented in the previous sections can be used to analyse the SHG-CD images. These images are shown in Fig. 4. For the longitudinal sample the values are centred on zero (0.02 ± 0.09) and the colour map appears dim, but the values still show a spatial non-random structure that is not solely due to noise. The distribution is symmetric around zero, showing that small deviation of fibre orientations occurs outwards on both sides of the image plane, according to Eqs (12) and (13). For non-zero cut angles, both positive and negative values are present in the SHG-CD images and the occurrence histograms show a binomial distribution with different amplitudes. This behaviour has been previously reported^42,44^. The SHG-CD images closely resemble phase images of the chiral ratio, which can be understood by looking at Eqs (12) and (13). The measured SHG-CD signals for (*α* ≠ 0) indicate that collagen fibres have a significant ${\chi }_{xyz}^{(2)}$ term. In addition, there is a phase difference, Δ, between ${\chi }_{xyz}^{(2)}$ and the non-chiral susceptibility tensor components. This is related to the presence of non-zero imaginary parts of the ${\chi }_{xyz}^{(2)}$ susceptibility, as shown in the DSMP measurements above.

Equation (12) and (13) show that the presence of both positive and negative values for SHG-CD can be due to collagen fibres of the same chirality but with opposite orientation (polarity). The assembly of collagen fibres of opposite polarity has been previously reported in studies using electron microscopy^50^, piezoelectric force microscopy^51,52^, interferometric SHG microscopy^53,54^ and polarimetric SHG microscopy with linearly polarised light^29^. This interpretation is further supported by the binomial distribution of SHG-CD values lying symmetrically from zero. In the transverse cut the positive and negative values are organised into islands, indicating that fibres have dominant orientation either parallel or anti-parallel with neighbouring fibres. The change in the polarity of the collagen fibrils changes the sign of the achiral *χ*^(2)^ tensor components^21^, while chiral tensor components preserve the same sign under rotation. Hence, the sign of SHG-CD can be used to determine the relative out-of-plane tilt angle of the fibres. On the other hand, the SHG-CD magnitude depends on the values of susceptibility components, the retardance between chiral and achiral components and the tilt angle *α*, so that interpretation is not straightforward and requires *a-priori* knowledge of 2 of the 3 aforementioned parameters.

In the previous section the values of ${\chi }_{zzz}^{(2)}/{\chi }_{zxx}^{(2)}$, ${\chi }_{xxz}^{(2)}/{\chi }_{zxx}^{(2)}$ and ${\chi }_{xyz}^{(2)}/{\chi }_{zxx}^{(2)}$ were extracted. In addition, it was shown that the achiral ${\chi }_{zzz}^{(2)}$, ${\chi }_{zxx}^{(2)}$ and ${\chi }_{xxz}^{(2)}$ components are in phase, which indicates that Δ_*zzz*,*zxx*_ = Δ_*xxz*,*zxx*_ = 0. On the other hand, the chiral component is not in phase with the other components. Using ${\chi }_{xxz}^{(2)}/{\chi }_{zxx}^{(2)}=1$, as validated above, and using the phase differences between chiral and each achiral components Δ_*zzz*,*xyz*_ = Δ_*zxx*,*xyz*_ = Δ_*xxz*,*xyz*_ = Δ, Eqs (12) and (13) can be simplified as:

14 $${\rm{SHG}} \mbox{-} {{\rm{CD}}}_{{\rm{simplified}}}=\tfrac{2|\tfrac{{\chi }_{xyz}^{(2)}}{{\chi }_{zxx}^{(2)}}|\sin (2\alpha )\cos (\alpha )\sin ({\rm{\Delta }})(|\tfrac{{\chi }_{zzz}^{(2)}}{{\chi }_{zxx}^{(2)}}|-3)}{{|\tfrac{{\chi }_{zzz}^{(2)}}{{\chi }_{zxx}^{(2)}}|}^{2}{\cos }^{4}(\alpha )+|\tfrac{{\chi }_{zzz}^{(2)}}{{\chi }_{zxx}^{(2)}}|(4{\cos }^{2}(\alpha )-6{\cos }^{4}(\alpha ))+8{|\tfrac{{\chi }_{xyz}^{(2)}}{{\chi }_{zxx}^{(2)}}|}^{2}{\sin }^{2}(\alpha )+9{\cos }^{4}(\alpha )-12{\cos }^{2}(\alpha )+8}$$

**Figure 5:**
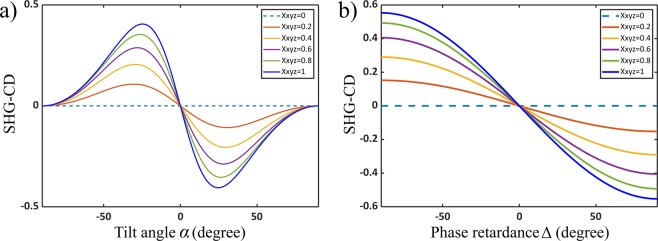
Magnitude of SHG-CD as a function of (**a**) the tilt angle, *α*, with Δ = *π*/4 and (**b**) the phase retardance between chiral and achiral susceptibility ratios, Δ, with *α* = 30°. They are plotted for different values of $|{\chi }_{xyz}^{(2)}|/|{\chi }_{zxx}^{(2)}|$, assuming $|{\chi }_{zzz}^{(2)}|/|{\chi }_{zxx}^{(2)}|=1.7$ and $|{\chi }_{xxz}^{(2)}|/|{\chi }_{zxx}^{(2)}|=1$.

The dependence of SHG-CD on the angle, *α*, and the retardance, Δ, for different values of chiral ratio can be examined. In Fig. 5a, Δ is fixed at *π*/4 and Eq. (14) is plotted for different values of ${\chi }_{xyz}^{(2)}$ as a function of *α*, showing that the SHG-CD is zero for fibres lying in or perpendicular to the image plane (*α* = 0° and 90°, respectively). Further, there are two values of *α* (±30°) for which the absolute value of SHG-CD is maximum. In Fig. 5b, *α* is fixed at 30° and Eq. (14) is plotted for different values of ${\chi }_{xyz}^{(2)}$ as a function of Δ. It can be seen that, for larger values of the phase retardation, the SHG-CD response is larger. In both graphs, it is seen that larger ${\chi }_{xyz}^{(2)}$ leads to larger absolute SHG-CD values. For collagen in the same tissue regions ${\chi }_{xyz}^{(2)}$ and Δ are expected to have only small variations, so that the main factors affecting the SHG-CD values stems from the polarity and tilt angle.

## Conclusion

All complex-valued observable *χ*^(2)^ laboratory-frame susceptibility components were extracted for collagen fibres in pig tendon cut at different orientation angles, and were further used to deduce the chiral, ${\chi }_{xyz}^{(2)}/{\chi }_{zxx}^{(2)}$, and achiral, ${\chi }_{zzz}^{(2)}/{\chi }_{zxx}^{(2)}$ and ${\chi }_{xxz}^{(2)}/{\chi }_{zxx}^{(2)}$, molecular susceptibility component ratios. The imaginary part of chiral ratio showed significant values, indicating retardance between the chiral and achiral molecular susceptibility components. The achiral molecular susceptibility component ratios had no retardance between each other. The imaginary contribution indicates a significant presence of magnetic dipole and/or quadrupole effects in the SHG imaging of collagen at off-resonance wavelengths. The imaginary components of ${\chi }_{xyz}^{(2)}$ are directly related to SHG-CD. The equation for the SHG-CD of thin biological tissues was derived and the values were found to be significant when three conditions are simultaneously met: (i) a non-zero out-of-image-plane fibre orientation angle, *α* ≠ 0, (ii) presence of chiral components, ${\chi }_{xyz}^{(2)}$, and (iii) a non-zero phase difference between the ${\chi }_{xyz}^{(2)}$ and the other susceptibility components. By assuming that collagen fibres have similar molecular susceptibilities at any particular wavelength in the same tissue, the SHG-CD can be interpreted as indicating out-of-image-plane tilt angle *α*, where positive and negative values represent opposite polarities.

Extraction of the molecular-ratio parameters, including the achiral and chiral ratio, and chiral amplitude and phase, provides a sensitive tool for ultrastructural characterisation of collagen. The collagen alterations are known to occur with diseases such as tumour initiation, progression and metastatic spread^4–14^. Further, the SHG-CD results can reveal the polarity of collagen fibres in the tissue. The polarity information can be used to investigate the piezoelectric and pyroelectric properties of collagen that are relevant for studying various tissues including bone and tendon^55–58^. SHG-CD can also be used as a method to investigate the 3D orientation of collagen in the sample. In addition, since SHG-CD has been already used as a new tool to differentiate normal from diseased tissues^23^, a better understanding of the underlying physical mechanisms of SHG-CD signals can help with interpretation of the results.

Therein lies the potential for translation of these findings into clinical impact as a means to further improve the diagnostic sensitivity and/or specificity of non-linear label-free histopathology in, for example, biopsied tissues.

## Data Availability

The datasets generated during and/or analysed during the current study are available from the corresponding author on reasonable request.

## References

[1] Wess TJ (2005). Collagen fibril form and function. Adv. Protein Chem..

[2] Nimni ME (1983). Collagen: structure, function, and metabolism in normal and fibrotic tissues. Semin. Arthritis Rheum..

[3] Ottani V, Raspanti M, Ruggeri a (2001). Collagen structure and functional implications. Micron (Oxford, Engl.: 1993).

[4] Provenzano PP (2006). Collagen reorganization at the tumor-stromal interface facilitates local invasion. BMC Medicine.

[5] Burke K, Tang P, Brown E (2013). Second harmonic generation reveals matrix alterations during breast tumor progression. J. Biomed. Opt..

[6] Golaraei A (2014). Characterization of collagen in non-small cell lung carcinoma with second harmonic polarization microscopy. Biomed. Opt. Express.

[7] Tokarz D (2015). Ultrastructural features of collagen in thyroid carcinoma tissue observed by polarization second harmonic generation microscopy. Biomed. Opt. Express.

[8] Golaraei A (2016). Changes of collagen ultrastructure in breast cancer tissue determined by second-harmonic generation double Stokes-Mueller polarimetric microscopy. Biomed. Opt. Express.

[9] Pupa SM, Ménard S, Forti S, Tagliabue E (2002). New insights into the role of extracellular matrix during tumor onset and progression. J. Cell. Physiol..

[10] Théret N (2001). Increased extracellular matrix remodeling is associated with tumor progression in human hepatocellular carcinomas. Hepatol. (Baltimore. Md.).

[11] Provenzano PP (2008). Collagen density promotes mammary tumor initiation and progression. BMC Medicine.

[12] Kalluri R (2003). Basement membranes: Structure, assembly and role in tumour angiogenesis. Nat. Rev. Cancer.

[13] Navab R, Strumpf D, To C, Pasko E, Kim K S, Park C J, Hai J, Liu J, Jonkman J, Barczyk M, Bandarchi B, Wang Y H, Venkat K, Ibrahimov E, Pham N-A, Ng C, Radulovich N, Zhu C-Q, Pintilie M, Wang D, Lu A, Jurisica I, Walker G C, Gullberg D, Tsao M-S (2015). Integrin α11β1 regulates cancer stromal stiffness and promotes tumorigenicity and metastasis in non-small cell lung cancer. Oncogene.

[14] Drifka, C. R. *et al*. Highly aligned stromal collagen is a negative prognostic factor following pancreatic ductal adenocarcinoma resection. *Oncotarget* **7**, In preparation, 10.18632/oncotarget.12772 (2016). 10.18632/oncotarget.12772 PMC5342807 27776346

[15] Roth S, Freund I (1979). Second harmonic generation in collagen. The J. Chem. Phys..

[16] Freund I, Deutsch M (1986). Second-harmonic microscopy of biological tissue. Opt. Lett..

[17] Tuer AE (2009). Three-Dimensional Visualization of the First Hyperpolarizability Tensor. J. Comput. Chem..

[18] Tuer AE (2011). Nonlinear optical properties of type I collagen fibers studied by polarization dependent second harmonic generation microscopy. The journal physical chemistry. The journal physical chemistry. B.

[19] Stoller P, Reiser KM, Celliers PM, Rubenchik AM (2002). Polarization-modulated second harmonic generation in collagen. Biophys. J..

[20] Stoller P, Kim B-M, Rubenchik AM, Reiser KM, Da Silva LB (2002). Polarization-dependent optical second-harmonic imaging of a rat-tail tendon. J. Biomed. Opt..

[21] Stoller P, Celliers PM, Reiser KM, Rubenchik AM (2003). Quantitative second-harmonic generation microscopy in collagen. Appl. Opt..

[22] Brasselet S (2011). Polarization-resolved nonlinear microscopy: application to structural molecular and biological imaging. Adv. Opt. Photonics.

[23] Chen X, Nadiarynkh O, Plotnikov S, Campagnola PJ (2012). Second harmonic generation microscopy for quantitative analysis of collagen fibrillar structure. Nat. Protoc..

[24] Tuer AE (2012). Hierarchical model of fibrillar collagen organization for interpreting the second-order susceptibility tensors in biological tissue. Biophys. J..

[25] Su PJ, Chen WL, Chen YF, Dong CY (2011). Determination of collagen nanostructure from second-order susceptibility tensor analysis. Biophys. J..

[26] Hu P-S (2012). Imaging of biological tissues with pixel-level analysis of second-order susceptibility. J. Biomed. Opt..

[27] Hu PS (2012). The use of second-order susceptibility as contrast mechanism for label-free imaging of biological tissue. IEEE J. on Sel. Top. Quantum Electron..

[28] Kumar R (2015). Polarization second harmonic generation microscopy provides quantitative enhanced molecular specificity for tissue diagnostics. J. Biophotonics.

[29] Golaraei Ahmad, Mirsanaye Kamdin, Ro Yeji, Krouglov Serguei, Akens Margarete K., Wilson Brian C., Barzda Virginijus (2018). Collagen chirality and three-dimensional orientation studied with polarimetric second-harmonic generation microscopy. Journal of Biophotonics.

[30] Adler E (1964). Nonlinear optical frequency polarization in a dielectric. Phys. Rev..

[31] Byers JD, Yee HI, Petralli-Mallow T, Hicks JM (1994). Second-harmonic generation circular-dichroism spectroscopy from chiral monolayers. Phys. Rev. B.

[32] Fischer P, Buckingham AD (1998). Surface second-order nonlinear optical activity. J. Opt. Soc. Am. B.

[33] Verbiest, T., Clays, K. & Rodriguez, V. *Second-order nonlinear optical characterization techniques: An introduction* (CRC Press, 2009).

[34] Samim M, Krouglov S, Barzda V (2015). Double Stokes Mueller polarimetry of second-harmonic generation in ordered molecular structures. J. Opt. Soc. Am. B.

[35] Samim M, Krouglov S, Barzda V (2016). Nonlinear Stokes-Mueller polarimetry. Phys. Rev. A - At. Mol. Opt. Phys..

[36] Kontenis L (2016). Second harmonic generation double stokes Mueller polarimetric microscopy of myofilaments. Biomed. Opt. Express.

[37] Huttunen Mikko J, Erkintalo Miro, Kauranen Martti (2009). Absolute nonlinear optical probes of surface chirality. Journal of Optics A: Pure and Applied Optics.

[38] Petralli-Mallow T, Wong TM, Byers JD, Yee HI, Hicks JM (1993). Circular dichroism spectroscopy at interfaces: A surface second harmonic generation study. J. Phys. Chem..

[39] Kauranen MM (1995). Chiral effects in the second-order optical nonlinearity of a poly(isocyanide) monolayer. Adv. Mater..

[40] Chen X, Raggio C, Campagnola PJ (2012). Second-harmonic generation circular dichroism studies of osteogenesis imperfecta. Opt. Lett..

[41] Campbell KR, Campagnola PJ (2017). Wavelength-Dependent Second Harmonic Generation Circular Dichroism for Differentiation of Col i and Col III Isoforms in Stromal Models of Ovarian Cancer Based on Intrinsic Chirality Differences. J. Phys. Chem. B.

[42] Chen M.-Y., Huttunen M.J., Kan C.-W., Deka G., Lin Y.-Y., Ye C.-W., Wu M.-J., Liu H.-L., Chu S.-W. (2018). Resonant nonlinear microscopy reveals changes in molecular level chirality in native biological tissues. Optics Communications.

[43] Campbell KR (2018). Polarization-resolved second harmonic generation imaging of human ovarian cancer. J. Biomed. Opt..

[44] Lee H (2013). Chiral imaging of collagen by second-harmonic generation circular dichroism. Biomed. Opt. Express.

[45] Huttunen MJ, Partanen M, Bautista G, Chu S-W, Kauranen M (2015). Nonlinear optical activity effects in complex anisotropic three-dimensional media. Opt. Mater. Express.

[46] Sandkuijl D, Tuer AE, Tokarz D, Sipe JE, Barzda V (2013). Numerical second- and third-harmonic generation microscopy. J. Opt. Soc. Am. B.

[47] Major A, Cisek R, Barzda V (2006). Femtosecond Yb:KGd(WO(4))(2) laser oscillator pumped by a high power fiber-coupled diode laser module. Opt. Express.

[48] Pena A-M (2006). Chiroptical effects in the second harmonic generation from collagens I and IV: Applications in nonlinear microscopy. Nonlinear Opt. Quantum Opt..

[49] Maki JJ, Kauranen M, Persoons A (1995). Surface second-harmonic generation from chiral materials. Phys. Rev. B.

[50] Parry DAD, Craig AS (1977). Quantitative electron microscope observations of the collagen fibrils in rat-tail tendon. Biopolymers.

[51] Harnagea C (2010). Two-dimensional nanoscale structural and functional imaging in individual collagen type i fibrils. Biophys. J..

[52] Denning D, Alilat S, Habelitz S, Fertala A, Rodriguez BJ (2012). Visualizing molecular polar order in tissues via electromechanical coupling. J. Struct. Biol..

[53] Rivard M (2014). Imaging the noncentrosymmetric structural organization of tendon with Interferometric Second Harmonic Generation microscopy. J. Biophotonics.

[54] Bancelin S (2016). Fast interferometric second harmonic generation microscopy. Biomed. Opt. Express.

[55] Fukada, E. *et al*. Related content piezoelectric effects in collagen. *Jpn. J. Appl. Phys*. **3** (1964).

[56] Cerrolaza M, Duarte V, Garzón-Alvarado D (2017). Analysis of bone remodeling under piezoelectricity effects using boundary elements. J. Bionic Eng..

[57] Ahn AC, Grodzinsky AJ (2009). Relevance of collagen piezoelectricity to “Wolff’s Law”: a critical review. Med. Eng. Phys..

[58] Lang SB (1966). Pyroelectric effect in bone and tendon [4]. Nature.

